# Maximal strength training as physical rehabilitation for patients with substance use disorder; a randomized controlled trial

**DOI:** 10.1186/s13102-016-0032-2

**Published:** 2016-03-31

**Authors:** Runar Unhjem, Grete Flemmen, Jan Hoff, Eivind Wang

**Affiliations:** Department of Circulation and Medical imaging, Faculty of Medicine, the Norwegian University of Science and Technology, Prinsesse Kristinas gt. 3, 7006 Trondheim, Norway; Department of Research and Development, Clinic of Substance Use and Addiction Medicine, St. Olav University Hospital, Trondheim, Norway; Department of Physical Medicine and Rehabilitation, St. Olav University Hospital, Trondheim, Norway; Division of Psychiatry, Department of Østmarka, St. Olav University Hospital, Trondheim, Norway; Department of Internal Medicine, University of Utah, Salt Lake City, Utah USA

**Keywords:** Muscle strength, One repetition maximum, Rate of force development, V-wave, Physical health, Mental health

## Abstract

**Background:**

Patients with substance use disorder (SUD) suffer from multiple health and psychosocial problems. Because poor physical capacities following an inactive lifestyle may indeed contribute to these problems, physical training is often suggested as an attractive supplement to conventional SUD treatment. Strength training is shown to increase muscle strength and effectively improve health and longevity. Therefore we investigated the feasibility and effect of a maximal strength training intervention for SUD patients in clinical treatment.

**Methods:**

16 males and 8 females were randomized into a training group (TG) and a control group (CG). The TG performed lower extremities maximal strength training (85-90 % of 1 repetition maximum (1RM)) 3 times a week for 8 weeks, while the CG participated in conventional clinical activities.

**Results:**

The TG increased hack squat 1RM (88 ± 54 %), plantar flexion 1RM (26 ± 20 %), hack squat rate of force development (82 ± 29 %) and peak force (11 ± 5 %). Additionally, the TG improved neural function, expressed as voluntary V-wave (88 ± 83 %). The CG displayed no change in any physical parameters. The TG also reduced anxiety and insomnia, while the CG reduced anxiety.

**Conclusion:**

Maximal strength training was feasible for SUD patients in treatment, and improved multiple risk factors for falls, fractures and lifestyle related diseases. As conventional treatment appears to have no effect on muscle strength, systematic strength training should be implemented as part of clinical practice.

**Trial regestration:**

ClinicalTrials.gov Identifier: NCT02218970 (August 14, 2014).

## Background

In addition to their drug abuse, patients with substance use disorder (SUD) suffer from multiple health and psychosocial comorbidities, resulting in a life expectancy 20–30 years less than the general population [1, 2]. Compared to the average population these patients are more frequently represented in medical care, with an elevated incidence of cardiovascular disease [1, 2], diabetes [1, 2], cancer [1, 2], suicide [1, 2], as well as traumas, falls and fractures [3–5]. Recent findings in our laboratory show that muscle strength and aerobic fitness are markedly reduced in SUD patients compared to healthy age-matched individuals [6]. Low muscle strength is associated with increased incidence of falls and fractures [7, 8], poor mechanical efficiency [9], elevated risk of cancer [10] and cardiovascular disease [11], and is even shown to be an independent predictor of all-cause mortality in both patient populations and healthy [12–14].

Strength training has become an increasingly common measure to improve muscle strength in different patient populations, and effectively reduce the risk of medical conditions and mortality. Maximal strength training, with heavy loads (>85 % of 1 repetition maximum (1RM)) and emphasis on intended concentric velocity has been successfully applied in multiple patient populations in our labs, and is shown to induce particularly large improvements in rate of force development (RFD) and muscle strength [15–19]. The improvements in maximal strength and RFD are suggested to predominantly rely on neural factors, with little or no change in body mass [9, 18, 20], which results in the training being even more suitable in populations where gains in weight are not sought after. Importantly, no injuries have been reported following these interventions, indicating that the training is not only effective, but also safe. Perhaps even more than the maximal strength, rapid force development is shown to be important for functional status, mechanical efficiency, balance adjustments and the prevention of falls and fractures [21–23]. Because the RFD relies mainly on neuromuscular properties [24], strength training applied to induce functional gain in patient populations should target neural adaptations. Assessed by the use of evoked reflex recordings, our research group has previously documented neural adaptations in both patient and healthy populations following maximal strength training [17, 25].

Although SUD patients are reported to have low muscle strength and aerobic capacity [6], there are few studies of systematic physical training as a part of clinical SUD treatment [26]. While physical activity is commonly used in conventional treatment [27], it appears not to apply a sufficient overload for taxing the muscular strength. Thus, maximal strength training would likely offer additional health benefits, and effectively reduce the risk of medical conditions. In addition to physical benefits, strength training is shown to have a positive effect on mental health, reducing anxiety and depression levels [28–30]. A low muscle strength has even been shown to independently be associated with an elevated rate of suicide [31]. In general, adherence to an exercise regime is also suggested to improve treatment outcomes and possibly reduce relapse rates in patients suffering from alcohol and substance abuse [26, 32].

Since physical activity in clinical treatment often appear random and unstructured [33], without the sufficient overload to produce gains in muscular strength, the aim of this study was to assess if a maximal strength training intervention was feasible for SUD patients, and would yield the previously documented beneficial physical and mental effects of such a training regime. We hypothesized that (1) SUD patients would be able to carry out the 8 week maximal strength training intervention, and (2) that the training group would improve maximal strength, RFD, efferent neural drive, depression, anxiety and insomnia more than the control group that participated in conventional treatment.

## Methods

### Subjects

**Fig. 1:**
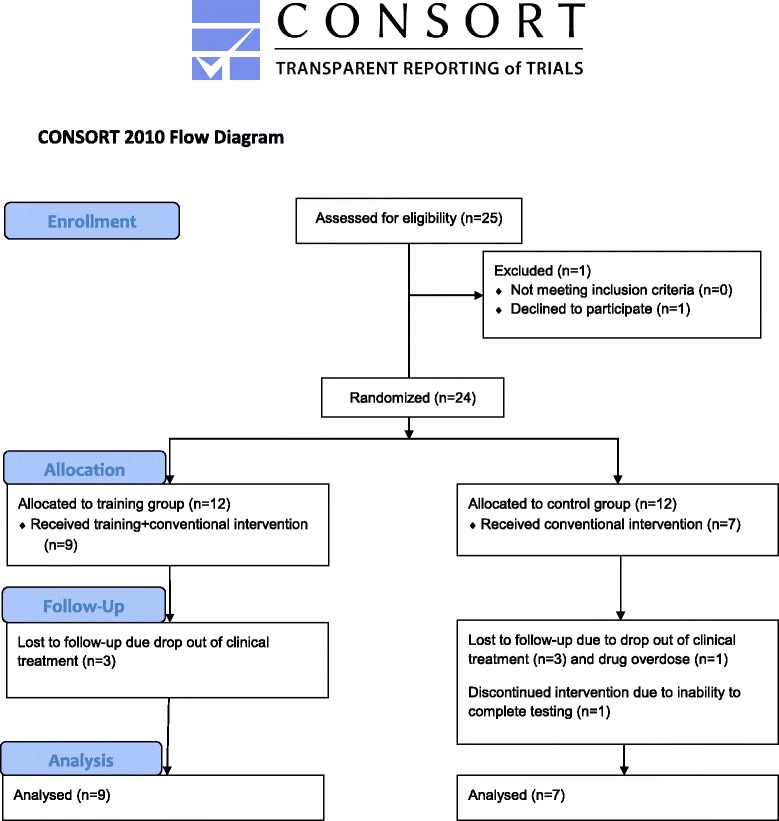
CONSORT flow diagram of study design

24 patients diagnosed with SUD, classified within ICD-10: F10-F19 (mental and behavioral disorders due to psychoactive substance use), were included in the study from February to March 2013. All subjects participated in a ~3 month residential long term treatment at a substance abuse clinic at the University hospital, and had amphetamine as their primary drug. After providing their informed consents subjects were randomized to either a maximal strength training group (TG) or a control group (CG) participating in conventional activities (Fig. 1). Subjects were assigned a number between 1 and 24, and randomization was performed using a publicly accessible official website designed for research randomization (https://www.randomizer.org). Subjects were excluded if they had been abstinent and/or systematically participated in strength training for the last six months. Other exclusion criteria were cardiovascular or respiratory disease, not being able to carry out the testing procedure or failure to participate in at least 20/24 training sessions. Patient characteristics and medical use are shown in Table 1. The study was approved by the regional ethical committee (REK-nord) and conducted in accordance with the declaration of Helsinki.

**Table 1:** Patient characteristics and medical use

	TG (*n* = 9)	CG (*n* = 7)	Combined (*n* = 16)
Men/Women (n)	6/3	7/0	13/3
Age (yr)	33 ± 9	29 ± 5	32 ± 8
Weight (kg)	80.2 ± 18.2	81.8 ± 9.6	80.9 ± 14.3
Height (cm)	173 ± 10	181 ± 5	177 ± 9
First time drug use (age)	14 ± 2	15 ± 2	14 ± 2
Duration of abuse (yr)	13 ± 10	11 ± 4	12 ± 8
Current Smoker	7	6	13
Primary drug:			
Amphetamine	9	7	16
Secondary drug:			
Alcohol	4	1	5
Cocaine		1	1
Cannabis	5	5	10
Symptoms for medicine prescription:			
ADHD	1	1	2
Allergies	3	4	7
Anxiety		3	3
Arthiritis	2		2
Asthma/COPD	3	1	4
Depression	3	1	4
Epilepsy		1	1
Hypertension	5		5
Schizofenia/Bipolar	4	1	5
Migrene	3		3
Substitutional treatment		1	1
Other	5	1	6

Data are presented as mean ± SD, *TG*; training group, *CG*; control group. Type of medication is reported on indication of symptoms according to common directory. The prescribed medicine in substitutional treatment is subuxone. Others: atherothrombosis, diabetes, infections

### Extent of drug use

To get an overview of the extent of drug use the first page of EuropASI was applied [34]. The index quantifies which substances the subject has used, age at first time drug use and years of use. Further the clinic provided information of prescribed medicine for the overall participating group of patients. Patient characteristics and medical use are given in Table 1.

### Testing procedure

All subjects conducted the testing procedure before and after the 8 week training intervention. On the day of testing, neuromuscular measurements (V-wave) were carried out first, followed by 1RM hack squat, 1RM plantar flexion and hack squat RFD. After the strength measurements, psychological questionnaires were filled out to assess levels of insomnia, anxiety and depression. Subjects were asked to not engage in any physical training on the day of testing or the day before.

### Strength measurements

One repetition maximum (1RM) was measured in hack squat and plantar flexion. Hack squat 1RM was obtained in a hack squat machine (Impulse Fitness IT7006, Shandong, China) angled 45° to vertical. For the plantar flexion test, the participants were seated in a calf rise machine (Impulse Health Tech IT7005, Shandong, China), with a knee joint angle of ~90°, and performed their lifts from an ankle joint angle of ~20° dorsiflexion in the lower position, up to ~30° plantar flexion in the upper position. Before testing the subjects were familiarized with the testing apparatus during an extensive warm up procedure, however no additional familiarization session was arranged. For both hack squat and plantar flexion, 1RM was achieved by increasing the load by 5-10 kg until the subject was not able to complete the lift. A three minutes rest was given between each trial, and correct joint angles were ensured. 1RM was achieved within 6–9 trials, and the highest load completed was recorded as 1RM.

RFD was recorded in the hack squat machine with a force platform at 2000Hz (9286AA, Kistler, Switzerland) attached to the foot plate. Each subject was given three attempts with a load corresponding to 80 % of pretest 1RM. Only the best trial was used for analyzes. The subjects were instructed to move slowly down to a knee joint angle of 90°, have a short stop to avoid eccentric action involvement, and then mobilize maximally in the concentric phase of the movement. Three minutes rest was given between each trial. The highest concentric force was recorded as peak force and RFD was calculated as ∆force between 10 % and 90 % of peak force [9].

### Neuromuscular measurements

Neuromuscular measurements were assessed by voluntary V-waves, with the subjects seated in a fixed version of the plantar flexion apparatus used for dynamic strength measurements. The V-wave method involves electrical stimulation of the tibial nerve, applied to evoke reflex potentials and motor potentials in afferent and efferent nerves. During supramaximal electrical stimulations all afferent and efferent nerve fibers are recruited simultaneously, and the reflex volley traveling the muscle spindle reflex circuit will collide with electrically evoked action potentials traveling antidromically in the efferent axons. Because of these collisions the reflex volley will be completely abolished during rest and not reach the muscle. In contrast, during maximal voluntary contraction (MVC) the efferent drive to the muscle will collide with the antidromic potentials, leaving some efferent axons open for transmission of the reflex. A higher efferent drive will clear more axons for reflex transmission, and will thus allow more of the reflex volley to pass through to the muscle, where it is recorded as a V-wave. Based on this, the amplitude of V-wave is used to express the efferent neural drive during MVC.

Reflex potentials were evoked by a current stimulator (DS7AH, Digitimer, Welwyn Garden City, UK), in the tibial nerve, in the popliteal fossa. The electrical current was delivered by gel-coated (Lectron 2 conductive gel, Pharmaceutical innovations INC, Newark, NJ, USA) bipolar felt pad electrodes, 25 mm between tips, 8 mm diameter (Digitimer, Welwyn Garden City, UK). The electrodes were held by hand throughout the testing procedure, and positioned at the site evoking the largest reflex amplitude. Evoked potentials were recorded through self-adhesive AG/AgCI electrodes (Ambu, M-00-S/50, Ballerup, Denmark) placed as recommended by SENIAM [35] on m. soleus. Before electrode attachment the skin was carefully preparated to minimize the inter-electrode impedance; impedance level <5 kΩ were required. To provide equal conditions from pre- to posttest, pictures were taken of the electrode placement at pretest, and used for identical positioning at posttest.

Searching for the maximal direct motor potential (M_max_) the current intensity was gradually increased by 2–5 mA until the M-wave reached a plateau. Between 70 and 180 mA was needed to evoke M_max_. To validate the M_max_ three supramaximal stimuli at 150 % of the current intensity needed to reach the plateau were given. Eight V-waves were evoked during MVC by delivering a supramaximal (150 %) stimulus at the point where the subject reached ~90 % of MVC force. Each MVC was separated by 1 min rest. Only V-wave recordings, in which the M-wave was > 90 % of M_max,_ were used for analyzes. The maximal V-wave amplitude (V_max_) was expressed relative to M_max_ (V/M-ratio), to allow between subjects comparisons. Changes in V/M-ratio are used to express changes in efferent drive following training.

### Psychological questionnaires

In addition to the physical testing two questionnaires were implemented; Insomnia Severity Index (ISI) to measure level of insomnia, and Hospital Anxiety & Depression Scale (HAD), used to estimate symptoms of anxiety and depression. These self-report questionnaires were answered in conjunction with the pre- and posttest of muscular strength, as measures of psychological changes during the period of the study. The ISI has been evaluated to be a clinically useful tool for screening and quantifying perceived insomnia severity [36]. It is composed of 7 items targeting different categories of sleep disturbance severity. The items are rated at a five-point Likert scale (0–4) summed up to provide a total score ranging from 0–28, where a higher score indicates more severe insomnia. The score categories are 0–7 (no clinically significant insomnia), 8–14 (subthreshold insomnia), 15–21 (clinical insomnia, moderate severity) and 22–28 (clinical insomnia, severe). The HAD self-assessment scale consists of a fourteen item scale, seven items relate to anxiety and seven relate to depression. On the seven item HADS subscales a score of 0–7 for either subscale is estimated within the normal range, a score of 11 or higher implies a probable presence of a mood disorder. A score of 8–10 is considered signs of a mood disorder [37].

### Training intervention

Both the TG and the CG attended the regular treatment program at the substance abuse clinic during the intervention period. The treatment program activities included: Ballgames (indoor-soccer, bandy and volleyball), yoga, stretching, outdoor walking, low resistance strength training (estimated <50 % of 1RM), ceramics, TV games and card games. Together this resulted in a total of ~3 h of physical activity per week. In addition, the TG received maximal strength training 3 times a week for a period of 8 weeks. The training intervention consisted of two exercises; hack squat and plantar flexion. Both exercises consisted of 4 sets of 4–5 repetitions, corresponding to 85-90 % of 1RM. The training load was increased with 5 kg if 5 repetitions were accomplished in the last set. Both exercises were conducted with a slow controlled movement in the eccentric phase, a short stop, and then maximal mobilization of force in the concentric movement. Hack squat was performed with 90° knee joint angle, while the plantar flexion exercise was performed from an ankle joint angle of ~20° dorsiflexion up to ~30° plantar flexion. Every training session was supervised to ensure proper technique and progression throughout the training period. While the TG participated in the supervised strength training, the CG chose to participate in self-elected supervised activities among the offered sports or games in the clinical treatment program.

### Statistical analyzes

Statistical analyzes were done using IBM SPSS Statistics 21 (Chicago, IL, USA), while figures were created using GraphPad Prism 5 (San Diego, USA). Independent and paired t tests were used to examine differences between groups at baseline and within groups following training, respectively. Between group differences following training were determined by use of two-way repeated ANOVAS. The Pearson test for linear regression was applied to assess correlations. Statistical significance level was set to *p* < 0.05. All variables exhibited normal distribution, as confirmed by quantile-quantile plots. Data are presented as mean ± SD unless otherwise noted.

## Results

### Completion

Of the 24 patients that were included in the study, 16 subjects completed the study period. 3 patients in the TG dropped out of the clinical treatment, and hence also dropped out of the study. In the CG 5 subjects dropped out; 3 patients dropped out of clinical treatment, 1 patient were not able to complete the testing procedure and 1 patient died from drug overdose. The withdrawal in the two groups resulted in an uneven distribution of genders, leaving no females in the CG at posttest. The participants in the TG adhered to 23 ± 1 of the 24 scheduled training sessions during the training period. The patients completed all commenced training sessions and the targeted intensity (85–90 % of 1RM) was reached in all sessions.

### Muscle strength measurements

**Fig. 2:**
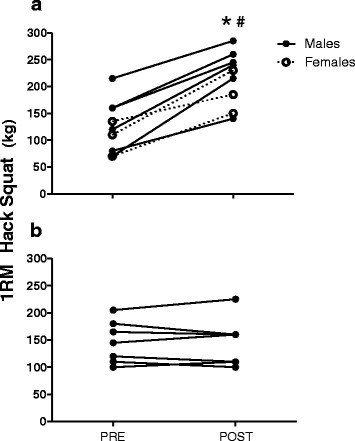
Hack squat one repetition maximum (1RM) for (**a**) the training group and (**b**) the control group from pre- to posttest. * *p* < 0.01, difference within group from pre- to posttest. # *p* < 0.01, difference between groups from pre- to posttest

**Fig. 3:**
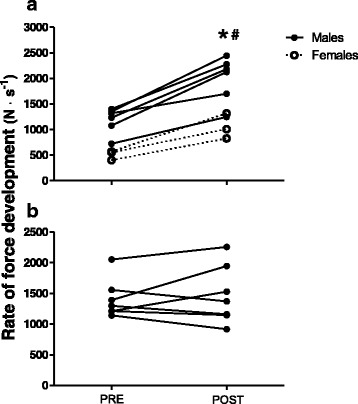
Hack squat rate of force development for (**a**) the training group and (**b**) the control group from pre- to posttest. * *p* < 0.01, difference within group from pre- to posttest. # *p* < 0.01, difference between groups from pre- to posttest

For the 16 subjects that completed the study, there was no significant difference between the TG and the CG in any of the measured strength parameters at pretest. After 8 weeks of maximal strength training the TG increased 1RM hack squat by 88 ± 54 % (*p* < 0.01) (Fig. 2), whereas plantar flexion 1RM increased from 98 ± 23 kg to 121 ± 17 kg (26 ± 20 %, *p* < 0.01). The TG also increased RFD by 82 ± 28 % (*p* < 0.01) (Fig. 3), whereas peak force increased from 1846 ± 357 N to 2045 ± 415 N (11 ± 5 %, *p* < 0.01). No significant changes were observed in the CG for any of the strength parameters.

### Neuromuscular measurements

**Fig. 4:**
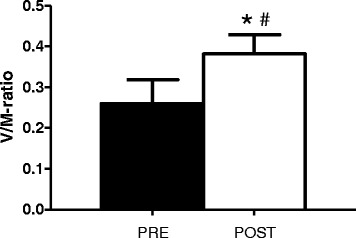
Data are presented as mean ± SE. Maximal V-wave/maximal M-wave (V/M-ratio) for the training group at pre- and posttest. * *p* < 0.01, difference within group from pre- to posttest. # *p* < 0.01, different from the control group from pre- to posttest

Maximal strength training led to an enhanced efferent neural drive in the TG. Following the 8 week training intervention the TG increased m. soleus V_max_ from 1583 ± 1596μv to 2189 ± 1375μv (92 ± 95 % (*p* < 0.01)). As there was no observed change in m. soleus M_max_ (6379 ± 2188μv vs. 6332 ± 2244μv), this resulted in an 88 ± 83 % (*p* < 0.01) increase in m. soleus V/M-ratio (Fig. 4). No significant changes were observed for the CG. Finally, ∆V/M-ratio correlated with ∆hack squat 1RM (r = 0.44, *p* < 0.05) and ∆plantar flexion 1RM (r = 0.57, *p* < 0.05).

### Psychosocial variables

Both the TG and the CG scored within “probable presence of mood disorder” at inclusion, with elevated scores of anxiety and insomnia. Following the study period both the TG and the CG displayed significant within group reductions in anxiety level (*p* < 0.05), while the level of insomnia significantly decreased only in the TG (*p* < 0.05) (Table 2). Also the level of depression tended to decrease in both groups (*p* = 0.11 for the TG and *p* = 0.10 for the CG). Neither of the within group differences were apparent as between-group differences.

**Table 2:** Psychological measurements, changes from pre- to posttest (scores from insomnia severity index and hospital anxiety and depression scale questionnaires)

	TG (*n* = 9)		CG (*n* = 7)	
	Pre	Post	Pre	Post
Anxiety (0–21)	12.3 ± 5.8	6.3 ± 3.9 *	11.1 ± 4.5	8.0 ± 4.8 *
Depression (0–21)	5.2 ± 2.1	3.0 ± 1.6	7.4 ± 4.7	4.9 ± 3.8
Insomnia (0–28)	9.2 ± 6.5	3.0 ± 2.0 *	13.3 ± 6.2	10.1 ± 5.3

Data are presented as mean ± SD, *TG*; Training group, *CG*; Control group. Score categories anxiety and depression: Normal (0–7); signs of mood disorder (8–10); probable presence of mood disorder (11–21). Score categories insomnia: No clinically significant insomnia (0–7); subthreshold insomnia (8–14); clinical insomnia, moderate severity (15–21); clinical insomnia, severe (22–28). * *p < 0.05*, difference within group pre- to posttest

## Discussion

### Main findings

SUD patients suffer from physical and psychological deconditioning as a consequence of their detrimental lifestyle. Since strength training is documented to improve both physical and mental health, we sought to investigate the feasibility and efficiency of a maximal strength training regime for a group of SUD patients in residential treatment. The main findings were that 1) A maximal strength training intervention was feasible for SUD patients in treatment, 2) Maximal strength training effectively improved maximal strength and muscle force development characteristics, likely caused by alterations in the central nervous system, 3) Anxiety and insomnia were improved following the clinical treatment period.

### Improved maximal strength and muscle force development characteristics

As expected the SUD patients that completed the strength training intervention displayed large improvements in all the measured strength parameters. The 88 % increase in hack squat 1 RM after 8 weeks of training is even somewhat higher than most previous maximal strength training studies, typically ranging between 25–45 % [9, 18, 19, 38, 39]. Since familiarization was included as a part of the training intervention in this study, this likely contributed to the large strength gain. Additionally, the very low baseline of the weakest subject, which allowed for a very large percentage improvement of ~200 %, also contributed to the high percentage improvement of the TG. Nevertheless, our findings demonstrate the large strength gain achievable when heavy loads and maximal intended concentric velocity are emphasized in strength training. Recognizing that the low physical baseline of the patients in the current study allows large training adaptations, both physiologically and mathematically, the large increase in hack squat 1RM highlights the clinical benefit of a high intensity training intervention in effective physical rehabilitation. The health benefits from an 88 % improvement in leg muscle strength are unquestionable. Ortega et al. [31] reported that Swedish men with high muscular strength had 35 % lower risk of developing cardiovascular disease, 15–65 % lower risk of having any psychiatric diagnosis and 20 % lower risk of all cause mortality when compared to men with low muscle strength. Also Ruiz et al. [10, 12] found the risk of mortality from cancer, cardiovascular disease and other causes to be inversely correlated with muscle strength. Both the Ortega et al. (2012) study and the Ruiz et al. [10, 12] studies emphasize that subjects with low and very low muscle strength particularly suffer an increased risk of medical complications. Considering this, increasing the strength of the weakest individuals would provide the largest health benefit. Although we did not compare our subjects with a reference group some of the patients in the current study stood out as particularly weak. Interestingly it was these patients who apparently seemed to benefit the most from the training. This visual observation was also reflected in the psychosocial questionnaires, where the three weakest subjects exhibited substantial improvements in the psychosocial variables following the training period.

High muscle strength is also associated with lower risk of falls and fractures [7, 8]. Moreland et al. [40] reported that subjects with low and very low muscle strength exhibited elevated risk of single and recurrent falling (Odds ratio: 1.31–5.06). Considering the high incidence of non-drug related hospitalizations among SUD-patients, typically including traumas, falls and fractures [3–5], it is likely that the improved muscle strength would have a preventive effect on these high injury- and hospitalization rates. Balance adjustments and fall prevention do not only require maximal strength; the ability of rapid muscle contractions is often just as important, since the time frame to avoid a fall is short [21, 22, 41]. Because strength training with heavy loads and maximal concentric mobilization is associated with large gain in explosive strength, maximal strength training is argued to be particularly beneficial to induce gain in motor function. The 82 % increase in RFD in the current study adds evidence of the large improvements in explosive strength following maximal strength training regimes, and is similar to previous reports from our research group [18, 38, 39].

### Neuromuscular alterations and maximal strength training

The ~ twofold increase in V/M-ratio highlights that neuromuscular changes largely contributed to the gain in muscle strength. Although there is agreement that training-induced changes in muscle strength relies on a combination of neuromuscular and anabolic adaptations [42], studies involving maximal strength training have often claimed that the improvements were mainly of neuromuscular origin, due to large improvements in 1RM and RFD, with no change in body weight [9, 18, 20]. Based on the comparable large improvements in V/M-ratio and maximal strength, as well as the lack of change in body weight, our findings are in line with this notion. It is unlikely that the low number of repetitions, and thus low anabolic effect, was sufficient to induce any significant muscle growth, while the heavy loads and maximal mobilization seems to be optimal for neural adaptations [20, 43]. The 88 % increase in V/M-ratio is slightly higher compared to other strength training studies, typically displaying improvements of 50–80 % [44, 45]. However, these interventions have been conducted with a lower training intensity than the current, consequently also resulting in smaller improvements maximal strength. Therefore, in combination, our findings and previous studies, exhibits corresponding improvements in neuromuscular adaptations and muscular strength. Specifically, the changes in V-wave amplitude in the current study likely reflects an enhanced efferent neural drive to the muscle, probably due to increased motor unit firing frequency and/or increased motoneuron recruitment [44, 46]. This is because a higher efferent drive would allow more of the electrically evoked reflex volley to pass through to the muscle, hence resulting in the increased amplitude of the V-wave.

### Feasibility of maximal strength training in substance use clinical treatment

The 75 % completion rate of the TG in the current study exemplifies that although maximal strength training may be considered strenuous, SUD patients are in general capable of engaging in physically demanding training regimes. To date there have been few studies examining intensive physical training in SUD patients, but we have recently shown that also intensive endurance training is feasible for this patient group [33]. In agreement with our findings from the endurance training study, the SUD patients reported no difficulties carrying out the strength training, and the targeted intensity (85–90 % of 1 RM) was reached in all commenced training sessions, without any reports of pain or discomfort. Importantly, most of the subjects that participated reported that they found the simple and robust training motivating, and that they enjoyed observing their own steady and impressing large progression throughout the study. Although SUD is commonly associated with high rates of nonattendance and relapse [47, 48], we experienced no issues regarding subject compliance and attendance to the scheduled training sessions. None of the participating subjects in clinical treatment dropped out solely from the training intervention. Despite being simple and time-efficient to carry out, the training intervention likely benefits from supervision from a trained professional to provide commitment to, and understanding of, the training regime. This notion is also in agreement with previous studies employing training interventions in SUD-patients [33, 49]. Notably, our experience involves only patients participating in residential treatment. It should therefore be considered that the same feasibility and completion rates may not apply for outpatients.

### Maximal strength training and psychosocial health

The SUD patients in the current study revealed significant signs of mood disorder at inclusion, reflected in elevated scores of anxiety and insomnia. The TG showed a reduction in both anxiety and insomnia scores, as well as a trend towards less depression. However, a reduction in depression following endurance training has previously been reported [33]. In combination, this is evidence that effective, intensive exercise training is not mentally harmful but, again, feasible. Since these improvements in this study are not significantly different from the CG it is difficult to conclude whether the mental health improvements were related to the clinical treatment itself or if they were a result of the improvements in muscle strength. Physical activity is in general shown to positively affect mental health [29], and it may therefore be that the mental health improvements are more related to physical activity performed by both groups, rather than the improvements in muscle strength. However, given the large beneficial effect of an improved physical capacity, and the substantial risk-reduction for diseases and thus likely improvement in quality of life, a clinical treatment including effective physical training should be advocated. Indeed, a close association between physical training and mental health has previously been reported [50–52]. Furthermore, it should be questioned whether self-reporting questionnaires that are not able to detect large training-induced decreases in risk of lifestyle-related diseases are good enough.

### Clinical considerations for effective physical training in clinical treatment

Recognizing the close relationship between physical capacities, life style related diseases and mortality [10, 53], it is likely that implementation of effective physical training as standard part of the treatment for SUD patients would decrease the high rates of non-drug related hospitalizations. This study shows that maximal strength training not only is feasible as a part of the treatment, it also has a large effect size and is time efficient. Previously we have shown similar findings for endurance training [33]. Adding to the arguments for implementation of effective physical training in the clinic is also the poor rehabilitation results observed in the CG participating in conventional physical activity. The current study observed that the muscular strength and force characteristics in the CG remained unchanged following the 8 week period. In a previous study similar observations were also reported for endurance capacity [33]. Although SUD patients suffer from many challenges, it is important to recognize that their physical health constitutes an important part of the overall health. Since muscle strength and aerobic capacity are known to be important contributors to the physical health, we would argue that strength- and endurance training should be carried out concurrently in clinical SUD treatment. Not only are these physical characteristics shown to be very low in SUD patients [6], today’s treatment also appears to have very limited, if any endurance and strength effects. Importantly, this study, as well as a recent endurance training study [33] suggests that effective strength- and endurance training regimes are feasible and safe to carry out within this patient group.

Interestingly, the dropout rate in the TG (3 subjects) in the current study was lower than in the CG (5 subjects). Again, a similar finding was documented following endurance training (3 subjects) vs. conventional treatment (5 subjects) [33]. It is also of importance that the three subjects that dropped out of this study dropped out of the general clinical treatment, and not solely the adherence to the maximal strength training intervention. In support of this notion, it has previously been suggested that participation and adherence to an exercise program may have a positive effect on the relapse rates during alcohol recovery [32]. In combination, these findings suggest that implementation of effective physical training will improve the patients’ physical health more than conventional treatment, and it is likely that it may also lead to gains in psychosocial health.

### Study limitations

The training-induced changes of the main physiological variables were statistical significant in this study. However, a larger sample size may have been beneficial for the psychosocial variables, or perhaps a replacement by more detailed psychosocial questionnaires. While this study exemplifies that high intensity strength training is effective and feasible in SUD treatment, it should be noted that all patients in the current study had amphetamine as their primary drug, and that they were all recruited from the same clinic. While the conventional treatment in this clinic did not have any effect on the physical variables, it cannot be excluded that other clinics may have more effective treatment programs. Similarly, it can also be questioned whether our results would have been different if we had included patients with other primary drugs than amphetamine. As both patient characteristics and clinical treatment programs may vary between clinics, future studies should aim to investigate the effect of effective physical training in multiple clinics, and also aim for larger sample sizes to target psychosocial variables and include patients with different primary drugs.

## Conclusion

This study shows that maximal strength training is a feasible, safe and effective method to improve muscle strength and function during SUD treatment. The large improvements in maximal strength and RFD that were observed following two months of training seemed to rely largely on neuromuscular adaptations. The improvements in physical health implies that the SUD patients have reduced their risk for traumas, falls and fractures, life style related diseases and all-cause mortality. Recognizing the poor physical condition of SUD patients, effective physical training, targeting muscle strength and aerobic capacity should be implemented in clinical treatment to improve physical and mental health.

## Abbreviations

1RM: one repetition maximum
CG: control group
HAD: hospital anxiety & depression scale
ISI: insomnia severity index
M_max_: maximal M-wave amplitude
MVC: maximal voluntary contraction
RFD: rate of force development
SUD: substance use disorder
TG: training group
V_max_: maximal V-wave amplitude
V/M- ratio: maximal V-wave amplitude / maximal M-wave amplitude
